# P-1181. Gepotidacin vs Standard Antibiotics: A Systematic Review and Meta-Analysis of Efficacy and Safety in Urogenital Infections

**DOI:** 10.1093/ofid/ofaf695.1374

**Published:** 2026-01-11

**Authors:** A S H E S H DAS, umama Alam, Debvarsha Mandal, Ali Naseem, Aparna Malireddi, Hrishikesh Kommu, Jana Al Jnainati, Mohammed Muzammil, Asim Ali Syed, Aashwin Kaushal, Kunal Gahlot, Hanisha Reddy Kukunoor, Sailesh I S Kumar, rithvika Badugu

**Affiliations:** KPC Medical College and Hospital , Kolkata, India, Kolkata, West Bengal, India; Khyber medical university, Peshawar, Northern Areas, Pakistan; Avalon University School of Medicine, Cypress, TX; King edward medical university lahore, Lahore, Punjab, Pakistan; Andhra Medical College, San Jose, California; JIPMER, Hyderabad, Puducherry, India; University of Milano-Bicocca, Bergamo, Lombardia, Italy; Deccan College Of Medical Sciences, Chicago, Illinois; Deccan College of medical sciences, Hyderabad, Telangana, India; Government Medical College Amritsar, Amritsar, Punjab, India; Vardhman Mahavir Medical College and Safdarjung Hospital, Bhilwara, Rajasthan, India; Maheshwara medical college and hospital, Mountain house, California; Madras medical college, birmingham, Alabama; gandhi medical college , secunderabad, Hyderabad, Telangana, India

## Abstract

**Background:**

Gepotidacin is a novel, a triazaacenaphthylene antibiotic which has been proven to be an effective therapeutic option for several urogenital infections including Neisseria gonorrhea (NG). Amidst the rising antimicrobial resistance, this offers a promising alternative to traditional antibiotics. This systematic review and meta-analysis evaluates its efficacy and safety in treating urogenital infections.

**Figure:**
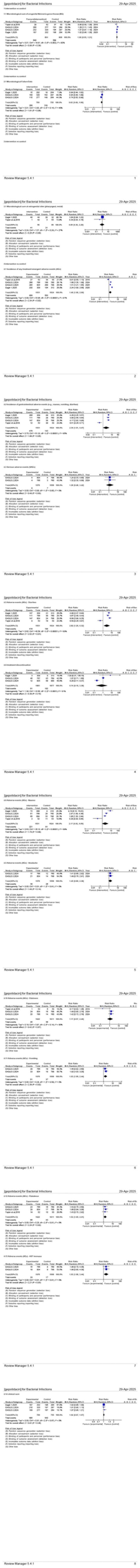

**Methods:**

A systematic search of PubMed, Embase, Scopus, and the Cochrane Library identified Randomized Controlled Trials (RCTs) comparing Gepotidacin with standard antibiotics for urogenital infections through March 2025. Data were analyzed using RevMan 4.2.1. Pooled risk ratios (RRs) with 95% CIs were calculated using Mantel-Haenszel methods. Fixed- or random-effects models were used based on heterogeneity (I²). Statistical significance was p < 0.05. Risk of bias was assessed via RoB 2.0.

**Results:**

Four RCTs (N = 3,855) were included. Gepotidacin achieved similar microbiological cure rates for urogenital NG vs. controls (RR 1.06; 95% CI, 0.93–1.21; *p* = 0.39; I² = 80%). Microbiological failure rates were lower but not significant (RR 0.83; 95% CI, 0.69–1.00; *p* = 0.05; I² = 49%). Extragenital site cure rates also showed no difference (RR 0.89; 95% CI, 0.36–2.20; *p* = 0.79; I² = 27%). Clinical cure rates were comparable (RR 1.02; 95% CI, 0.97–1.07; *p* = 0.50; I² = 0%). However, Gepotidacin was linked to a higher rate of treatment-emergent Adverse Events (AEs) (RR 1.46; 95% CI, 1.06–2.00; *p* = 0.02; I² = 91%), notably gastrointestinal AEs like diarrhea (RR 2.82; 95% CI, 1.26–6.32; *p* = 0.01; I² = 93%) and nausea (RR 2.17; 95% CI, 0.92–5.09; *p* = 0.08; I² = 89%). AST elevations were also more frequent (RR 1.93; 95% CI, 1.08–3.44; *p* = 0.03; I² = 0%).

**Conclusion:**

Gepotidacin demonstrates comparable efficacy to standard antibiotic treatment options for NG and other urogenital infections. However, it is associated with a higher incidence of gastrointestinal and hepatic adverse events which could possibly result in lower patient compliance.

**Disclosures:**

All Authors: No reported disclosures

